# Reply to Comment on “UGT2B17 modifies drug response in chronic lymphocytic leukaemia”

**DOI:** 10.1038/s41416-020-1006-4

**Published:** 2020-07-24

**Authors:** Chantal Guillemette, Michèle Rouleau, Katrina Vanura, Éric Lévesque

**Affiliations:** 1 Faculty of Pharmacy, Pharmacogenomics Laboratory, Centre Hospitalier Universitaire de Québec (CHU de Québec) Research Center, Laval University, Québec city, QC Canada; 2 Canada Research Chair in Pharmacogenomics, Québec city, QC Canada; 3 Division of Hematology and Hemostaseology, Department of Medicine I and Comprehensive Cancer Center, Medical University of Vienna, Vienna, Austria; 4 Faculty of Medicine, CHU de Québec Research Centre, Department of Medicine, Laval University, Québec city, QC Canada

**Keywords:** Oncology, Predictive markers, Haematological cancer

We have read with great interest the correspondence from Papamichos and Jungbauer, which further extends the conclusion of our study.^1^ Their observations suggest a potential role of human endogenous-retroviral sequences (HERVs) in the transcriptional control of the *UGT2B17* gene that would also drive its aberrant expression in lymphoid cancer cells.

UGT2B17 is the major UGT metabolic enzyme expressed in B-cells of chronic lymphocytic leukaemia (CLL) patients.^1,2^ Its expression influences cancer cell behaviour but also drug metabolism and response.^1,3,4^ The clinical relevance of *UGT2B17* as an RNA-based marker and an independent prognostic marker of survival is well supported.^2,3,5^ Our recent study further suggested a drug-induced expression of UGT2B17 that can lead to lesser anti-leukaemic response including to fludarabine and ibrutinib.^1^ However, the regulation of the *UGT2B17* gene in B-cells and the mechanism of its induction by drugs remain largely undefined.

As we showed, the expression of *UGT2B17* is driven by alternative promoters comprising exons 1b and 1c in lymphoid cell models and in CLL patients (Fig. 1a).^1^ Findings by Papamichos and Jungbauer, based on bioinformatic analyses, open a new perspective by raising the possibility that these sequences would be evolutionarily derived from HERVs acting as potential enhancers of *UGT2B17* transcription in CLL (Fig. 1b). A similar genetic setup was reported by Papamichos and co-authors in 2017 for multiple myeloma, suggesting a more general mechanism of UGT2B17 overexpression dependent on the transcriptional state of a HERV.^6^ Previous studies support an oncogenic role for HERVs in CLL.^7,8^

**Fig. 1:**
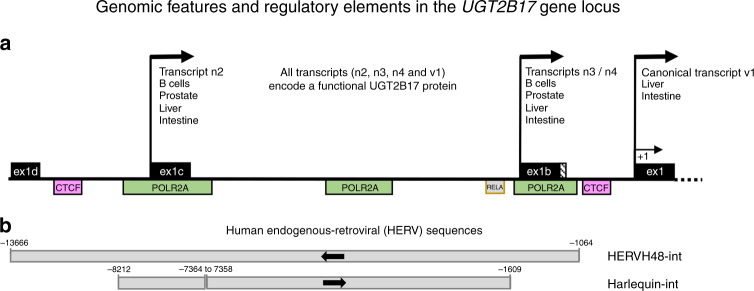
Schematic representation the *UGT2B17* gene locus summarising our recent observations and those of Papamichos and Jungbauer. **a** Expression of the UGT2B17 enzyme is driven by the canonical (v1) and alternative (n2, n3, n4) transcripts in multiple tissues. *UGT2B17* transcripts expressed in B-cells of CLL patients include novel exons (ex) 1c and 1b and are driven by alternative promoters, whereas hepatic transcripts are driven predominantly by the canonical promoter and exon 1. Note that all transcripts include the canonical exon 1, whereas exons 1b, 1c and 1d are mutually exclusive.^1,4,9^ Exon E0 described by Papamichos et al.^6^ corresponds to exon 1c. Positions of CTCF, POLR2A and RELA binding sites are based on UCSC datasets as described by Papamichos and Jungbauer. **b** The HERVs reported by Papamichos and Jungbauer based on datasets included on the UCSC human genome browser are positioned relative to the first *UGT2B17* exons. The sense of the HERVs are indicated by arrows. HERV48-int is in the reverse orientation relative to the *UGT2B17* gene and encompasses all novel exons including exon 1d, which is included in transcripts identified in the liver, intestine and/or prostate tissues.^9^ The Harlequin-int sequence includes exon 1c and sequences upstream of exon 1b. Coordinates are relative to the translational *UGT2B17* start site in exon 1 (+1) and were derived from the UCSC Annotation release 105.20190906 described by Papamichos and Jungbauer accessed on the UCSC Genome browser on 24 June 2020. CTCF CCCTC-binding factor; POLR2A RNA polymerase II; RELA p65 subunit of the NF-κB complex.

We would like to point out that our prior observations indicate that *UGT2B17* alternative transcripts are also expressed in normal tissues,^4,9^ suggesting that these putative HERV-derived sequences still retain expression capacity in normal cells. Therefore, although a rise in UGT2B17 expression in lymphoid cancer cells may be driven by an altered epigenetic landscape of HERVs possibly arising from cellular transformation, it is also plausible that these sequences acquired through evolution have enabled the remarkable tissue-specific expression of UGT2B17.

Our findings have revealed that prior knowledge of hepatic regulation of UGT2B17 expression, mainly derived from the canonical promoter, is not relevant to its expression in B-cells and other cancer cells.^1,4^ We suggested an involvement of the NF-κB pathway for its lymphoid expression.^1^ Papamichos and Jungbauer provide further evidence that supports our findings for a potential RELA (p65, NF-κB subunit) binding site embedded within the HERVs at the *UGT2B17* locus (Fig. 1a).

It remains to be experimentally demonstrated whether these HERVs play a role in the regulation and shaping of UGT2B17 expression in lymphoid and other cancer cells. Clarifying the in vivo implications of UGT expression and enzyme activity over disease course, induction of UGT expression upon drug treatment and the impact on drug response are also warranted. These aspects are part of our ongoing investigations to gain more insights into how the UGT pathway might impact cancer progression and drug response,^10^ with the goal to optimise cancer patient care.

## Data Availability

UCSC Genome Browser (https//genome.ucsc.edu/), *UGT2B17* gene locus.
